# A RNA nanotechnology platform for a simultaneous two-in-one siRNA delivery and its application in synergistic RNAi therapy

**DOI:** 10.1038/srep32363

**Published:** 2016-08-26

**Authors:** Mihue Jang, Hee Dong Han, Hyung Jun Ahn

**Affiliations:** 1 Center for Theragnosis, Biomedical Research Institute, Korea Institute of Science and Technology, Seoul, South Korea; 2 Department of Immunology, School of Medicine, Konkuk University, Chungju, South Korea

## Abstract

Incorporating multiple copies of two RNAi molecules into a single nanostructure in a precisely controlled manner can provide an efficient delivery tool to regulate multiple gene pathways in the relation of mutual dependence. Here, we show a RNA nanotechnology platform for a two-in-one RNAi delivery system to contain polymeric two RNAi molecules within the same RNA nanoparticles, without the aid of polyelectrolyte condensation reagents. As our RNA nanoparticles lead to the simultaneous silencing of two targeted mRNAs, of which biological functions are highly interdependent, combination therapy for multi-drug resistance cancer cells, which was studied as a specific application of our two-in-one RNAi delivery system, demonstrates the efficient synergistic effects for cancer therapy. Therefore, this RNA nanoparticles approach has an efficient tool for a simultaneous co-delivery of RNAi molecules in the RNAi-based biomedical applications, and our current studies present an efficient strategy to overcome multi-drug resistance caused by malfunction of genes in chemotherapy.

RNA, as macromolecular biomaterials, has intrinsic characteristics at the nanometer scale, and a variety of secondary structure motifs and loops found in RNA may provide powerful building blocks for fabrication of nanostructures^1^. Thus, RNA nanotechnology has been intensively investigated for creation of numerous therapeutic and biological nanostructures, and particularly in the treatment of cancer, genetic dysregulation and viral infection^2^. Using bottom-up assembly, we have reported that RNA nanoparticles (RNA NPs) containing multiple copies of a single siRNA sequences could systemically deliver siRNA to tumors and achieve sequence-specific gene silencing. In these studies, RNA polymers, generated by rolling circle transcription, were designed to partially form RNA/RNA or RNA/DNA double helices while single-stranded RNA regions increased the flexibility of RNA polymers, and finally, the base-pairing between RNA and cholesterol-conjugated DNA could condensate RNA polymer without the aid of polycationic reagents, generating nano-sized RNA particles^3^.

To further expand versatility of these RNA nanoparticles in the therapeutic applications, the next goal was to develop a two-in-one RNA NPs delivery system that simultaneously delivers two types of siRNAs for potent synergistic therapies based on RNAi. As regards chemotherapy failure in the cancer treatment, the intrinsic or acquired drug resistance of cancer cells is often affected by multiple gene pathways that are highly interdependent, and eventually reduces the cytotoxic effects of anticancer drugs, resulting in a relapse of cancer^4^. When the mechanisms of chemoresistance on most multi-drug resistance (MDR) cancers are classified into pump and non-pump resistance, the pump resistance, caused by membrane proteins such as P-glycoprotein (P-gp/MDR1/ABCB1)^5^,^6^,^7^,^8^,^9^, multidrug resistance protein (MRP, MRP-1/ABCC1)^10^,^11^,^12^, and breast cancer resistant protein (BCRP, ABCG2)^8^, deports drugs out of cells, whereas the non-pump resistance mainly activates the cell death defense *via* anti-apoptotic proteins such as BCL2, MCL1, and BCL-X_L_^13^,^14^,^15^,^16^.

To overcome such a chemoresistance using RNAi therapy, the simultaneous co-delivery of two types of siRNAs may be required in the combination therapy of MDR cancer, because the simultaneous gene silencing for both pump and non-pump resistance is required, rather than a single gene silencing, specific only for either pump or non-pump. However, when considering the combination therapy of anticancer drugs with simultaneous silencing of two genes, there still exist several hurdles to be addressed for the best synergistic outcome. Firstly, two types of siRNAs should be delivered *via* a two-in-one delivery system, not separately, to achieve the spatial synchronization. When the two siRNAs are delivered separately, only 10% of the cancer cell population will embrace one type of siRNA, and another 10% will embrace the other type of siRNA. Unless the two siRNAs delivery events are mutually coupled, the number of the cells that receive both types of siRNAs will be 1% (=10% × 10%) of the cancer cell population. However, two-in-one delivery allows the result to be the same 10% of cells now embracing both types of siRNAs. When compared to two separate deliveries, two-in-one delivery can thus enhance the efficiency of delivering two types of siRNAs to the same cell population by at least one order of magnitude. Secondly, an exceptionally high cellular cytotoxicity, expected from the combined therapy of drugs and siRNA, requires a strict tumor-specific delivery system to lower the risk of severe adverse effects in the healthy tissues^17^. Thirdly, each silencing in the simultaneous silencing for two different genes should not be attenuated in comparison with a separate single gene silencing, and particularly, the drug uptake should not be decreased even when both genes are silenced^18^. Additionally, there still remain many problems for delivering siRNA to tumors *in vitro* or *in vivo*, including the tricky cellular uptake, low loading efficacy for siRNA cargo, unsafety in the immune system, instability in bloodstream, and lack of targeting ability^19^.

We here report a RNA nanotechnology platform for synthesizing RNA NPs containing multiple tandem repeats of two types of siRNA sequences, *via* rolling circle transcription (RCT) and cholesterol-mediated RNA condensation. When both MDR1 and BCL2 siRNA sequences, chosen as one of the anticipated biomedical applications, were incorporated into the same RNA NPs, we could achieve the simultaneous gene silencing of both pump and non-pump resistance while addressing various siRNA delivery issues. Our advanced design of DNA templates for RCT allows polymeric, two types of RNAi molecules to be easily assembled into RNA NPs, achieving an increase in cargo loading. Particularly, the modified one-step condensing/ligand-displaying process allowed the particle size of RCT products to be precisely modulated by adjusting the complexing ratio with ligand-cholesterol-conjugated DNA, leading to favorable physicochemical features for efficient therapeutic applications. Also, the resulting RNA NPs showed the cancer-specific cellular uptake in a folate receptor-dependent way. Consequently, our RNA nanotechnology platform provides a tool of generating precisely controlled and biocompatible RNA NPs that can simultaneously silence both pump and non-pump resistance genes.

Using a two-in-one RNA NPs delivery system on MDR KB-V1 cancer cells, we examined the cytotoxicity effects from the simultaneous or sequential administration of RNA NPs and doxorubicin. MDR KB-V1 cancer cells, when pretreated with RNA NPs before doxorubicin administration, showed a marked increase in the intracellular concentration of doxorubicin, as well as a significant decrease in IC_50_ value of doxorubicin. Our studies indicate that our RNA platform method for a simultaneous two-in-one siRNA delivery system has great potential in RNAi-based therapeutic applications, for example, for overcoming multi-drug resistance in the cancer treatment.

## Results

### Design and synthesis of Dsi RNAtr/FA-DNA-CHOL complexes

**Figure 1:**
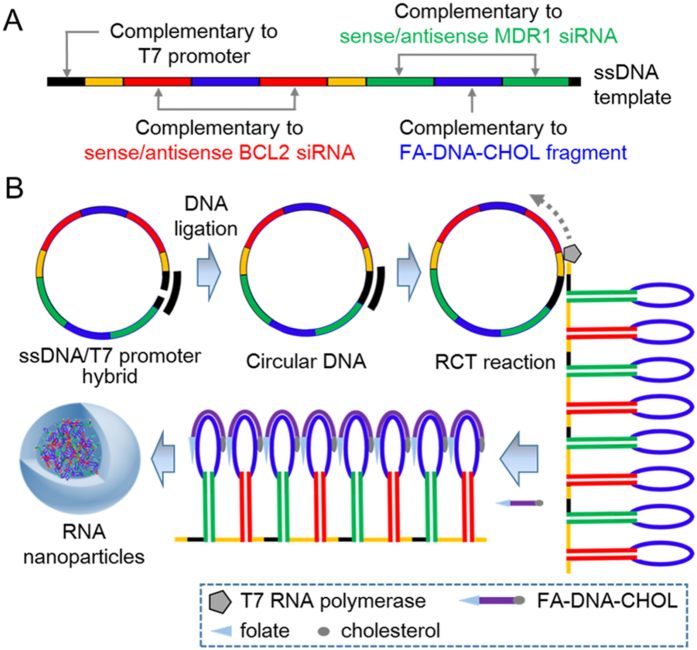
Schematic illustration demonstrating synthesis of RNA nanoparticles as a two-in-one siRNA delivery system. (**A**) Rational design of ssDNA template for co-delivery of MDR1 siRNA and BCL2 siRNA. (**B**) Schematic illustration demonstrating self-assembly of Dsi RNA nanoparticles (Dsi RNPs) *via* rolling circle transcription (RCT) and one-step hybridization with folate-DNA-cholesterol fragments (FA-DNA-CHOL). RNA transcripts polymerized by T7 RNA polymerase-mediated RCT reaction consist of multiple tandem repeats of RNA hairpins, which contain the alternate sequences of BCL2 and MDR1 siRNA. Through base pairing between RNA and DNA, FA-DNA-CHOL fragments are sparsely tethered to RNA transcripts, and consequently, the amphiphilic RNA transcripts/FA-DNA-CHOL complexes are able to self-assemble into the highly packed RNA nanoparticles, which are feasible for co-delivery of MDR1 and BCL2 siRNA to the same cancer cells.

To synthesize the two-in-one RNA nanoparticles for simultaneous co-delivery of MDR1 siRNA (siMDR1) and BCL2 siRNA (siBCL2), we first designed linear DNA template, which could be transcribed into RNA transcripts *via* RCT reaction, in a way that both siRNA sequences alternated with each other (Fig. 1A and Supplementary Table S1). The linear single-stranded DNA template was composed of sense and antisense BCL2 siRNA sequences, sense and antisense MDR1 siRNA sequences, loop regions in hairpin structures, linker regions between hairpins, and T7 promoter primer-binding sites (Fig. 1A). After both ends of linear DNA template were annealed with T7 primers using the complementary sequences, the nicked sequences were connected by T4 DNA ligase (Fig. 1B). Using T7 RNA polymerase-mediated RCT reaction against the resulting closed DNA template, we generated RNA transcripts (Dsi RNA transcripts or Dsi RNAtr), of which multiple tandem repeats of RNA hairpins contained the alternate sequences of siMDR1 and siBCL2. Next, we base-paired Dsi RNA transcripts with FA-DNA-CHOL fragments, which were obtained as a result of conjugation between folate (FA) and cholesterol-modified DNA (DNA-CHOL), and consequently, generated Dsi RNAtr/FA-DNA-CHOL complexes. The single stranded RNA sequences, repeatedly located between short hairpin RNA structures, were designed to be feasible for cleavage by dicer enzyme after cellular uptake.

### Characterization of Dsi RNA nanoparticles

**Figure 2:**
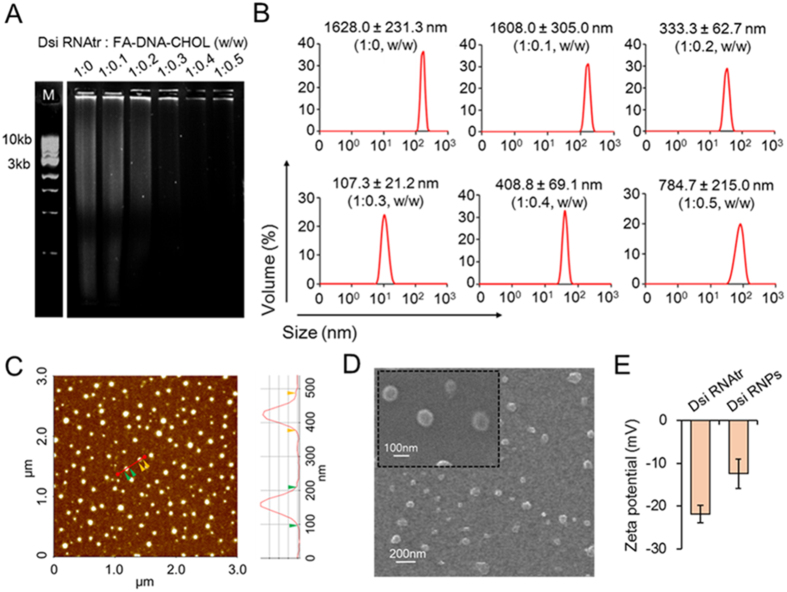
Characterization of RNA nanoparticles before or after base pairing of RNA transcripts with FA-DNA-CHOL fragments. (**A**) Gel retardation assay showing the base-pairing of FA-DNA-CHOL fragments to Dsi RNA transcripts (Dsi RNAtr) at the weight ratio indicated. Oligonucleotides were visible under UV irradiation after EtBr staining. (**B**) Hydrodynamic size distribution profiles of Dsi RNAtr/FA-DNA-CHOL complexes at the weight ratio indicated. (**C**) Atomic force microscopy (AFM) imaging of Dsi RNAtr/FA-DNA-CHOL complexes (Dsi RNPs). A cross-sectional profile along red line in the AFM images is denoted on the right side. (**D**) Field emission-scanning electron microscopy (FE-SEM) images demonstrating size and shape of Dsi RNPs. Inset shows high-magnification images of particles. (**E**) Zeta potential of Dsi RNAtr and Dsi RNPs determined by electron light scattering (ELS). The results are shown as the mean ± s.d. (n = 3).

Agarose electrophoretic analysis demonstrated that the molecular weights of Dsi RNA transcripts covered a wide range, but their base-pairing with FA-DNA-CHOL fragments formed the high molecular weight of assembled structures, and over a certain complexing ratio such as 1:0.3 (Dsi RNAtr:FA-DNA-CHOL, w/w), the assembled RNA complexes did not move down from the wells (Fig. 2A). In the dynamic light scattering analysis, the naked Dsi RNA transcripts showed about 1.6 μm of hydrodynamic diameter, but base-pairing of Dsi RNA transcripts with FA-DNA-CHOL conjugates allowed them to form the condensed particles (Fig. 2B). As the relative amounts of FA-DNA-CHOL increased, the hydrodynamic diameter of Dsi RNAtr/FA-DNA-CHOL complexes decreased, and consequently, at a complexing ratio of 1:0.3 (w/w), they showed the minimal diameter of 107.3 ± 21.2 nm, whereas overweighted FA-DNA-CHOL fragments, when exceeding a ratio of 1:0.3 (w/w), resulted in the formation of high molecular weight of aggregates, corresponding to more than 400 nm of diameter. These data suggest that the particle size of Dsi RNA transcripts can be precisely modulated by adjusting the complexing ratio with FA-DNA-CHOL.

As mentioned in the previous studies^20^, the amphiphilic properties of RNA complexes, acquired by sparsely tethering the hydrophobic cholesterol to Dsi RNA transcripts, allow the micrometer-sized Dsi RNA transcripts to be highly condensed into the nanometer-sized Dsi RNA particles (Dsi RNPs) in the aqueous solution. Particularly, the modified one-step condensation/folate-displaying protocol simplified the synthesis processes for RNA NPs. Moreover, atomic force microscopic analysis demonstrated that Dsi RNA nanoparticles had the sphere nanostructures with an average size of 109 ± 0.42 nm (Fig. 2C). Field emission-scanning electron microscopy (FE-SEM) also exhibited that Dsi RNPs were highly compact and had the sphere shape with an average size of 98.6 ± 1.01 nm (Fig. 2D). Therefore, we chose the optimal complexing ratio as 1:0.3 (w/w) in the present studies. It is worthy of note that when simultaneous silencing for two gene pathways is required to elicit various therapeutic effects, our facile condensation method can incorporate multiple tandem copies of double siRNA molecules into Dsi RNA nanoparticles simply by adjusting the ratio of FA-DNA-CHOL and Dsi RNA transcripts. Based on the static light scattering, Mw of Dsi RNPs was determined about 22400 kDa (Supplementary Fig. S1). Also, zeta potential measurement showed that the negative surface charge of Dsi RNPs was moderately weakened than that of Dsi RNA transcripts (Fig. 2E). The residual magnesium ions, which were supplemented during preparation process to be finally 10 mM, can compensate for the negative charge of oligonucleotide complexes^21^, and therefore the densely packed Dsi RNPs are expected to have more marked shielding effect than Dsi RNA transcripts, due to the higher surface density.

### Enhanced stability of Dsi RNPs in serum and innate immunogenicity studies

We further examined the efficacy of Dsi RNPs with respect to RNases attack in serum, dicer processing, and immunostimulatory response to address potential therapeutic applications of our RNA nanotechnology platform. When each of Dsi RNA transcripts and Dsi RNPs were mixed with FBS-containing solution, Dsi RNA transcripts were rapidly broken down to 20% within 30 m, whereas Dsi RNPs showed the enhanced stability, and thus, even at 6 h post-incubation, about 57.5% of Dsi RNPs remained intact (Supplementary Fig. S2). These results indicate that Dsi RNPs are much more stable against nucleases than Dsi RNA transcripts, because the highly condensed structures are less accessible to nucleases attack than the loosely packed ones. The naked monomeric siRNA was completely degraded within 30 m (data not shown). In contrast to the resistance to enzymatic degradation in serum, Dsi RNPs, which contain a number of RNA hairpins, need to be converted into the active siRNA molecules through the action of dicer enzyme after cellular internalization^22^. When we examined whether useful amounts of Dsi RNPs could be digested into siRNA *in vitro*, about 33.8% of the starting materials were converted to siRNA (corresponding to each of siMDR1 and siBCL2) within 24 h (Supplementary Fig. S3). Considering that siRNA cargo was loaded into Dsi RNPs with 37.3% of loading efficacy (see Supplementary Information), the dicer cleavage efficacy *in vitro* is estimated as 90.6%.

It has been reported that the exogenous RNA or RNA/DNA hybrids, including nucleic acid-based therapeutic agents, may cause the undesirable immunostimulatory response in primary human blood cells by stimulating the inflammatory cytokines closely associated with innate immunogenicity^23^,^24^,^25^,^26^. Since release or production of proinflammatory cytokines such as INF-α and IL-6 is often measured to predict immunostimulatory response of biomaterials^27^, we examined the release of both cytokines using human peripheral blood mononuclear cells (PBMCs). Based on the sandwich ELISA kit, neither Dsi RNA transcripts nor Dsi RNPs stimulated INF-α release at 24 h post-treatment, whereas monomeric siRNA complexed with polyethylenimine (PEI), a widely used gene transfection reagent *in vitro* and *in vivo*, induced the release of INF-α to a significant extent (Supplementary Fig. S4). Moreover, we did not observe the release of IL-6 on the Dsi RNPs-treated human PBMCs.

### Folate receptor-selective cellular binding and uptake of Dsi RNPs

**Figure 3:**
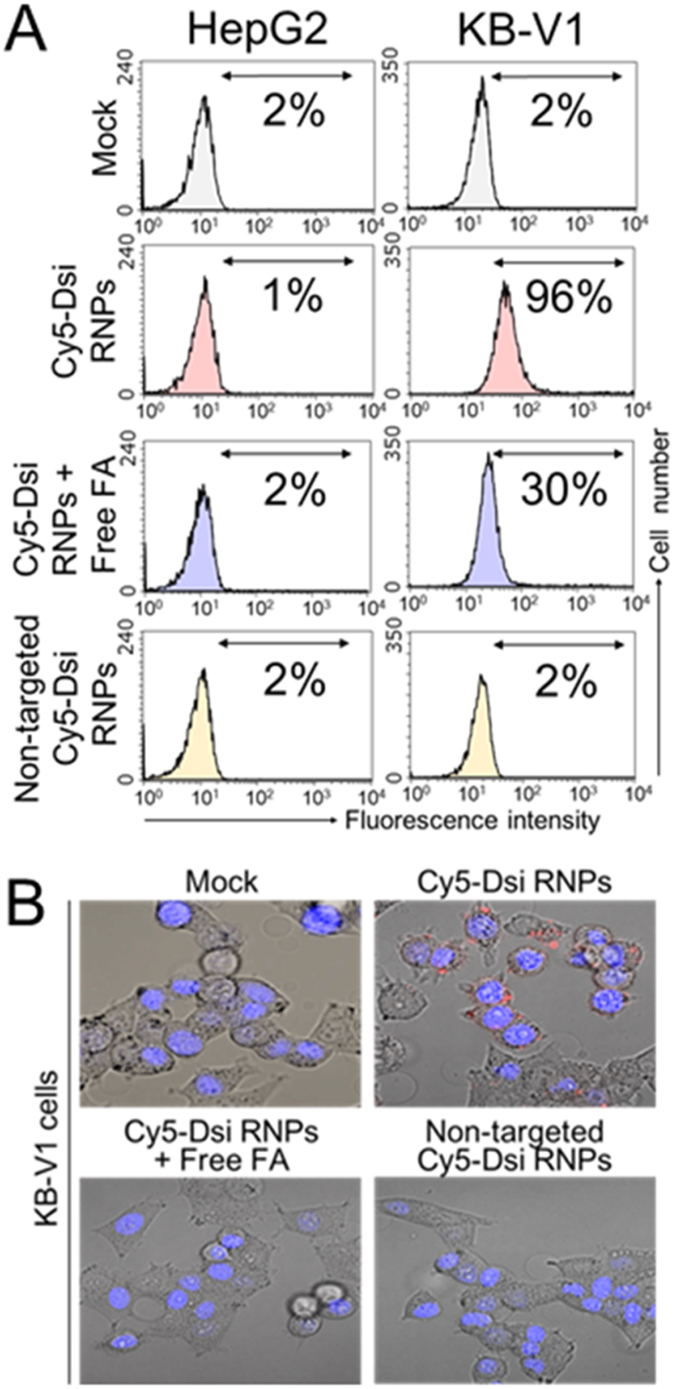
Cellular binding and internalization of Dsi RNPs in a FR-dependent manner. (**A**) Folate receptor (FR)-selective cell binding of Cy5-labeled Dsi RNPs in FR-negative HepG2 liver carcinoma cells and FR-positive KB-V1 cervix carcinoma cells, based on Flow cytometry. The percentage of cells sorted within a prefixed gate region for Cy-5 fluorescence is represented. Non-targeted Dsi RNPs could be obtained by using folate free DNA-CHOL fragments, instead of FA-DNA-CHOL fragments. For competition studies, folate receptors were blocked with free folate before Dsi RNPs treatment. (**B**) Cellular internalization of Cy5-labeled Dsi RNPs using confocal microscopic images. Red and blue signals indicate Cy5-labeled Dsi RNPs and DAPI dyes, respectively.

Compared with the normal cells, most cancer cells of endothelial origin have the characteristics that folate receptor (FR) is aberrantly overexpressed, and thus folate ligand, a naturally existing biomaterial with no immunogenicity, has been widely used for tumor-selective targeting^28^,^29^,^30^,^31^. Using flow cytometry analysis, we investigated whether Dsi RNPs selectively bound to cancer cells in a FR-dependent way. When the fluorescence dye Cy5-labeled Dsi RNPs were treated to the FR-positive KB-V1 cervical cancer cells, the fraction of Cy5-positive KB-V1 cells was approximately 96% at 3 h post-treatment, whereas the Cy5-positive HepG2 lung cancer cells were rarely found (Fig. 3A). The difference in the cellular binding of Dsi RNPs to each of cell lines can be explained by the relative amounts of folate receptors expressed on each cell line, as shown in the western blotting studies (Supplementary Fig. S5). When we partially blocked the folate receptors on the KB-V1 cancer cells with free folate ligands prior to the treatment of Cy5-labeled Dsi RNPs, the fraction of Cy5-labeled Dsi RNPs substantially decreased down to 30%. Also, Cy5-labeled folate-free Dsi RNPs (corresponding to non-targeted RNA nanoparticles) could hardly show any cellular binding to KB-V1 cells. As a control, HepG2 cells did not show any cellular binding for the Cy5-labeled folate-free Dsi RNPs, and also, the folate-pretreated HepG2 cells did not bind with Cy5-labeled Dsi RNPs.

In the confocal microscopic images for measurement of cellular uptake, Cy5-labeled Dsi RNPs exhibited the intracellular punctuate signals within KB-V1 cells at 3 h post-treatment, which indicated that Dsi RNPs were substantially internalized into the cells, while Cy5-labeled folate-free Dsi RNPs showed little signal (Fig. 3B). Also, KB-V1 cells, when saturated with free folate ligands, did not show any cellular uptake of Dsi RNPs. All things considered, these results imply that FA-displayed Dsi RNPs selectively bind to KB-V1 cancer cells *via* folate-folate receptor interactions, and subsequently internalized into cancer cells. Although the negative surface charge of Dsi RNPs, as shown in the Fig. 2E, does not allow Dsi RNPs to readily cross the anionic cell membrane through passive diffusion, the folate receptor-mediated cellular binding of Dsi RNPs are expected to increase their uptake on the cancer cells especially with the overexpressed folate receptors, as opposed to the folate-free Dsi RNPs.

### Quantitative determination of MDR1 and BCL2 mRNA after combination therapy of Dsi RNPs and doxorubicin

On many types of MDR carcinoma cell lines, it is reported that P-gp proteins, responsible for pump type of drug resistance, are intrinsically overexpressed or their expression is robustly induced in response to chemotherapeutics treatment^8^,^32^. Consistent with these reports, our western blotting analysis revealed that P-gp proteins were more overexpressed in MDR KB-V1 cell line than in their KB parent cell line (Supplementary Fig. S6). To examine the window of time when the drug resistance phenotypes are effectively suppressed and thus the MDR cells become sensitized to drugs, we treated MDR KB-V1 cells in two different approaches, either a two-step Dsi-24h-Dox-24h sequential or a Dsi/Dox-48h simultaneous method (see EXPERIMENTAL SECTION). As a control, we also treated MDR KB-V1 cells with siMDR1 RNPs (composed of MDR1 shRNA and scrambled BCL2 shRNA), siBCL2 RNPs (composed of BCL2 shRNA and scrambled MDR1 shRNA), or Dsc RNPs (composed of both scrambled shRNAs for MDR1 and BCL2).

**Figure 4:**
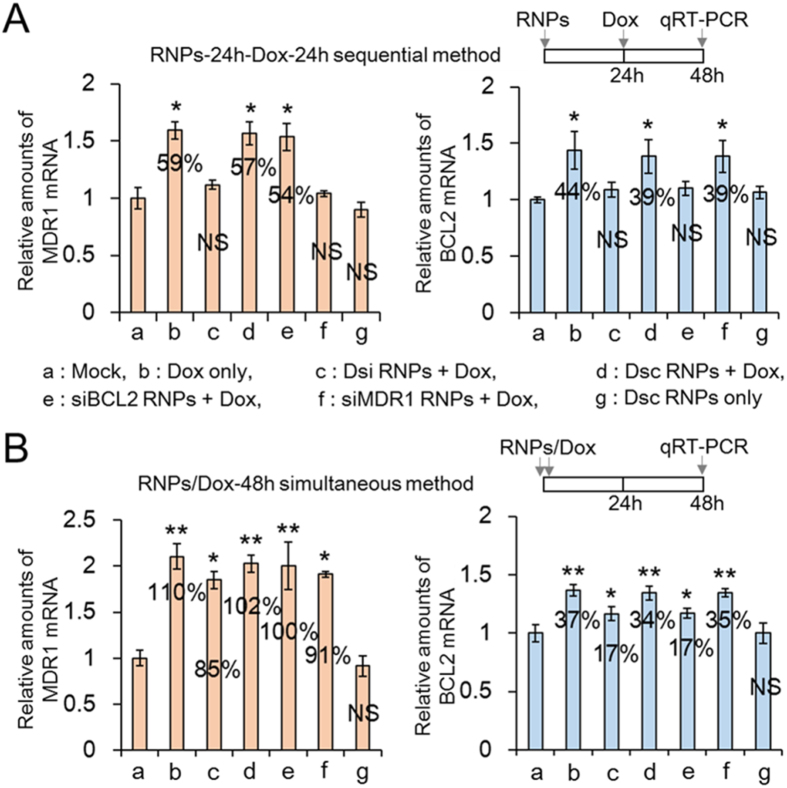
Quantitative determination of MDR1 and BCL2 mRNA on KB-V1 cells treated sequentially or simultaneously with various RNA nanoparticles and doxorubicin. (**A**) MDR1 and BCL2 mRNA measured by qRT-PCR after two-step RNPs-24h-Dox-24h sequential treatment. MDR KB-V1 cells were pretreated with RNA nanoparticles, and 24 h later, sequentially treated with doxorubicin. qRT-PCR was carried out 24 h after doxorubicin treatment. For clarity, this administration strategy is called “RNPs-24h-Dox-24h sequential method”. (**B**) MDR1 and BCL2 mRNA measured by qRT-PCR after RNPs/Dox-48h simultaneous treatment. MDR KB-V1 cells were treated with RNA nanoparticles and doxorubicin at the same time, and 48 h later, qRT-PCR was carried out. This administration strategy is called “RNPs/Dox-48h simultaneous method”. The alphabetic characters (a–g) represent the same formulations as those in the Figure A. The results are shown as the mean ± s.d. (n = 5). *p < 0.001, **p < 0.005 by one-way ANOVA with Tukey’s multiple comparison test, as compared to the mock control. NS = Not Significant (**A**,**B**).

First, we carried out qRT-PCR, which could quantify variation in each of MDR1 and BCL2 mRNA level on the MDR KB-V1 cells. When treating MDR KB-V1 cells with doxorubicin alone, we observed a large increase in MDR1 mRNA level by 59–110%, as well as a large increase in BCL2 mRNA level by 37–44% (Fig. 4A,B). However, the doxorubicin administered sequentially 24 h after Dsi RNPs treatment (that is, a two-step Dsi-24h-Dox-24h sequential treatment) did not show any noticeable increase in the relative mRNA level of MDR1 and BCL2 respectively, on the basis of an overall ANOVA statistics. In contrast with this successful suppression, the simultaneous treatment of doxorubicin and Dsi RNPs (that is, a Dsi/Dox-48h simultaneous treatment) failed to suppress the rapid increase in each mRNA level and thus led to an 85% and 17% of increase in each mRNA level. This failure in gene suppression may be attributed mainly to the rapid activation of MDR1 and BCL2 gene by the function of doxorubicin before Dsi RNPs trigger mRNA degradation, whereas the two-step Dsi-24h-Dox-24h sequential treatment may allow sufficient time to degrade both mRNA before doxorubicin induces the activation of both genes (Supplementary Fig. S7).

Next, we compared the silencing efficiency of our two-in-one Dsi RNA nanoparticles with that of siMDR1 RNPs or siBCL2 RNPs, which were responsible for the separate MDR1 or BCL2 gene silencing. siMDR1 RNPs, when treated in the siMDR1-24h-Dox-24h sequential method, significantly suppressed the rapid increase of MDR1 mRNA while not breaking down BCL2 mRNA. However, siMDR1 RNPs, when simultaneously treated with doxorubicin (siMDR1/Dox-48h), resulted in a 91% of increase in MDR1 mRNA level. siBCL2 RNPs, when treated in the siBCL2-24h-Dox-24h sequential method, remarkably kept down the rapid increase of BCL2 mRNA without degrading MDR1 mRNA, whereas siBCL2 RNPs simultaneously treated with doxorubicin (siBCL2/Dox-48h) led to a 17% of increase in BCL2 mRNA level. Therefore, Dsi RNPs for simultaneous silencing of both genes showed to effectively suppress each of BCL2 and MDR1 genes in comparison with siBCL2 RNPs or siMDR1 RNPs (Fig. 4A,B). On the other hand, the scrambled Dsc RNPs, when pretreated to MDR KB-V1 cells 24 h before doxorubicin, did not suppress the rapid increase of either MDR1 or BCL2 mRNA, and thus each mRNA increased to a significant extent, as shown in the free doxorubicin-treated control cells.

### Suppression of MDR1 and BCL2 protein expression after combination therapy of Dsi RNPs and doxorubicin

**Figure 5:**
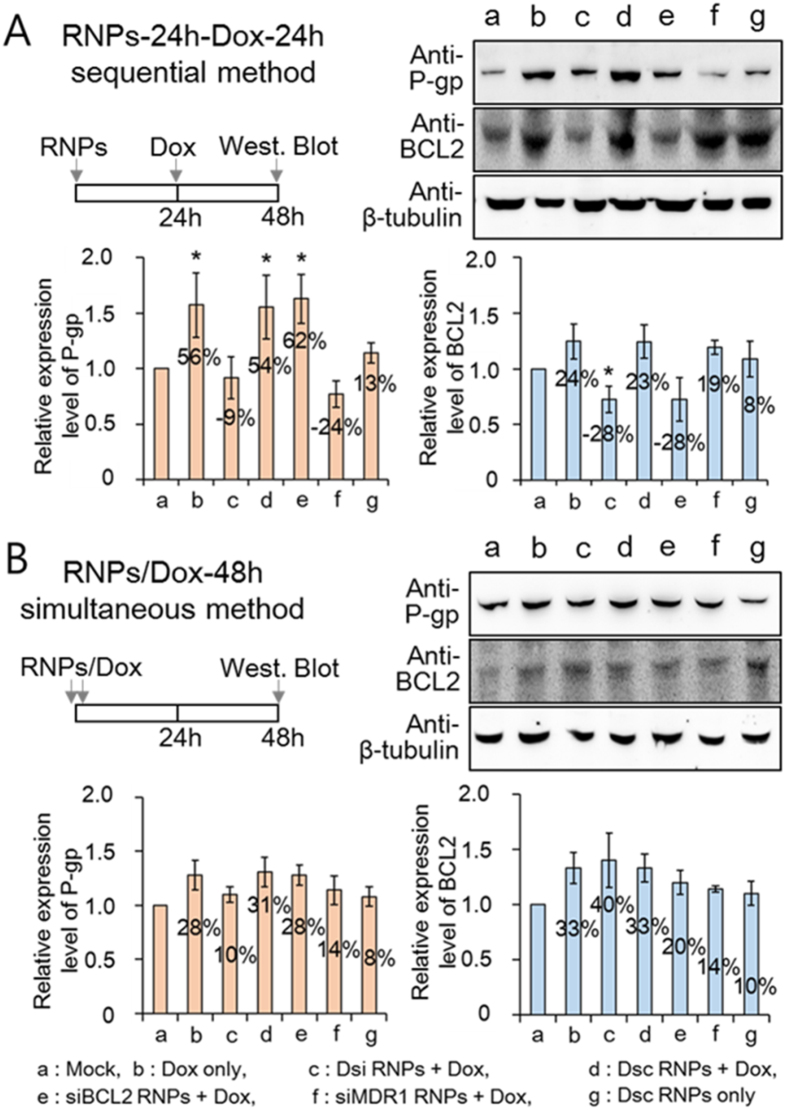
Quantitative determination of MDR1 and BCL2 protein on KB-V1 cells. Suppression of MDR1 and BCL2 protein expression on KB-V1 cells treated sequentially or simultaneously with various RNA nanoparticles and doxorubicin. Immunoblotting and quantitative measurements of P-gp and BCL2 proteins on KB-V1 cells treated with various RNA nanoparticles and doxorubicin in either two-step RNPs-24h-Dox-24h sequential method (**A**) or RNPs/Dox-48h simultaneous method (**B**). Relative amounts of P-gp or BCL2 proteins on the sample cells were plotted relative to those in the averaged values of control cells (mock). The results are shown as the mean ± s.d. (n = 5). *p < 0.01 by one-way ANOVA with Tukey’s multiple comparison test, as compared to the mock control.

To more validate the silencing efficiency of Dsi RNPs, we carried out western blotting using anti-P-gp and anti-BCL2 antibodies. As reported in a wide range of MDR cancers^33^,^34^, we observed a large increase in both the P-pg and BCL2 protein levels on the free doxorubicin-treated KB-V1 cells, in comparison with those on the control KB-V1 cells (Fig. 5A,B). However, the doxorubicin administered sequentially 24 h after Dsi RNPs treatment (Dsi-24h-Dox-24h) did not increase the amount of either P-gp or BCL2 proteins, and consequently, both proteins levels were significantly decreased by 9% and 28% respectively, when compared with each protein level on the control KB-V1 cells. Considering the significantly elevated protein levels for P-pg and BCL2 on the free doxorubicin-treated KB-V1 cells, such a dramatic decrease in each protein levels demonstrates that both pump and non-pump phenotypes are efficiently suppressed. In contrast, the simultaneous treatment of doxorubicin and Dsi RNPs (Dsi/Dox-48h) revealed a large increase in the P-gp and BCL2 protein levels by 10% and 40% respectively, which implies that this treatment strategy cannot efficiently suppress either pump or non-pump phenotype. Thus, these results indicate that Dsi RNPs, when pretreated to MDR cells, can efficiently suppress the MDR phenotypes.

As a separate silencing control, siMDR1-24h-Dox-24h and siBCL2-24h-Dox-24h treatment remarkably decreased the P-gp and BCL2 protein levels by 24% and 28% respectively, without silencing other phenotype. In contrast, each simultaneous treatment including the siMDR1/Dox-48h and siBCL2/Dox-48h treatment led to an increase in the P-gp and BCL2 protein levels by 14% and 20% respectively. Both the sequential and simultaneous treatment of the scrambled Dsc RNPs and doxorubicin, as a negative control, did not suppress either P-gp or BCL2 proteins, and thus led to a significant increase in each of the P-gp and BCL2 protein levels, as shown in the only free doxorubicin-treated cells. Therefore, these results demonstrate that Dsi RNPs as a two-in-one siRNA delivery system can simultaneously suppress both types of drug resistance without loss of efficacy.

### Suppressed drug efflux on Dsi RNPs-pretreated KB-V1 cells

**Figure 6:**
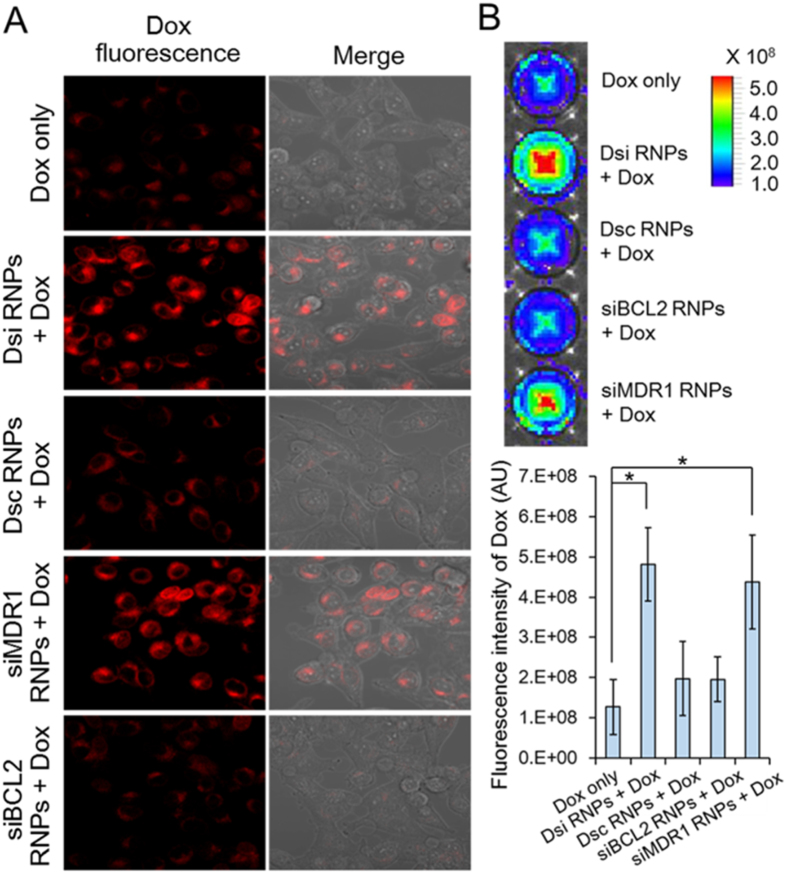
Intracellular localization of doxorubicin on KB-V1 cells pretreated with RNA nanoparticles. (**A**) Confocal microscopic images showing the intracellular localization of doxorubicin in MDR KB-V1 cells. Following the RNPs-24h-Dox-24h method, MDR KB-V1 cells were pretreated with various RNA nanoparticles, and 24 h later, treated with doxorubicin. Confocal microscopic images were obtained 12 h after doxorubicin treatment. Red signals represent the fluorescence emitted from doxorubicin. (**B**) Quantitative measurement of intracellular doxorubicin concentrations on the RNPs-pretreated KB-V1 cells using Xenogen IVIS Lumina imaging system and Image J software. MDR KB-V1 cells on 96-well plates were treated in the RNPs-24h-Dox-24h method, and 12 h later, NIRF images were measured. Fluorescence intensities of intracellular doxorubicin were also plotted. The results represent the mean ± s.d. (n = 5). *p < 0.01 by one-way ANOVA with Tukey’s multiple comparison test, as compared to the Dox only control.

Next, we examined accumulation of doxorubicin on the Dsi RNPs-treated KB-V1 cells using confocal microscopy. The free doxorubicin-treated KB-V1 cells showed a weak cellular accumulation of doxorubicin as a result of drug efflux, a characteristic in the MDR cancer cells (Fig. 6A). Similarly, KB-V1 cells treated with the scrambled Dsc RNPs or siBCL2 RNPs 24 h prior to doxorubicin administration (Dsc-24h-Dox-24h or siBCL2-24h-Dox-24h) revealed a weak cellular accumulation of doxorubicin due to the lack of suppression of MDR1 function. However, KB-V1 cells treated with Dsi RNPs or siMDR1 RNPs 24 h prior to doxorubicin administration (Dsi-24h-Dox-24h or siMDR1-24h-Dox-24h) exhibited a significantly increased accumulation of doxorubicin because the silencing of MDR1 gene inactivated the drug efflux function of P-gp protein. Furthermore, we measured the concentration of the intracellular doxorubicin on MDR KB-V1 cells by using a fluorescence imaging system. Neither the scrambled Dsc RNPs nor siBCL2 RNPs treatment exhibited any detectable increase in concentration of the intracellular doxorubicin, as shown in the free doxorubicin-treated KB-V1 cells (Fig. 6B). However, KB-V1 cells treated with either Dsi RNPs or siMDR1 RNPs 24 h prior to doxorubicin administration showed a remarkable increase. By the same token, these results demonstrate that Dsi RNPs, two-in-one siRNA delivery carriers, suppress the function of P-gp protein as much as siMDR1 RNPs, single siRNA delivery carriers, can do, without loss of efficacy in gene silencing, and also indicate that doxorubicin accumulation in MDR cells, which is highly associated with cell cytotoxicity, can be enhanced by Dsi RNPs treatment.

### Induction of apoptosis after Dsi-24h-Dox-24h sequential treatment

**Figure 7:**
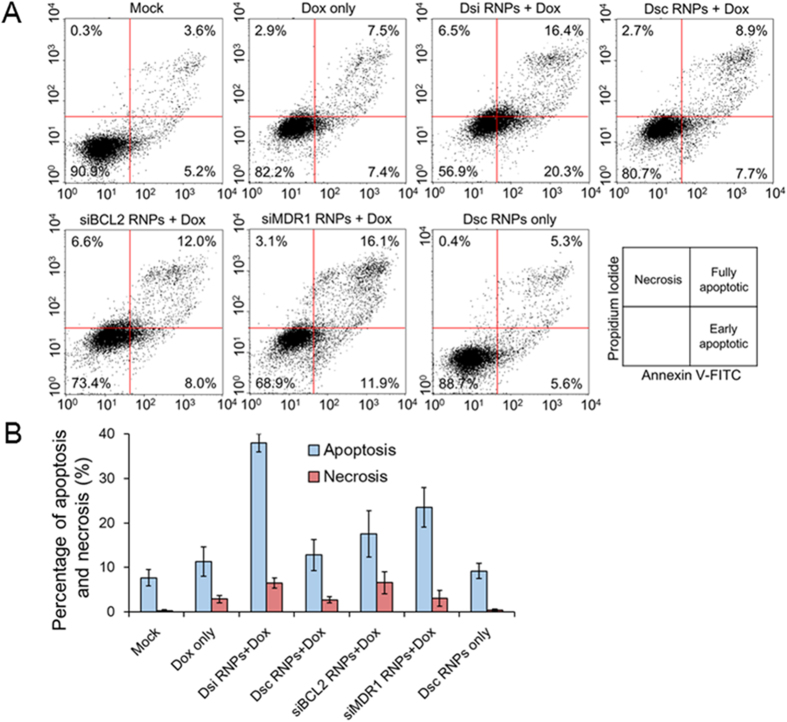
Induction of apoptosis on MDR KB-V1 cells after two-step Dsi-24h-Dox-24h sequential treatment. (**A**) MDR KB-V1 cells were treated with various RNA nanoparticles and doxorubicin in the two-step RNPs-24h-Dox-24h sequential method, and then induction of apoptosis was examined by flow cytometry analysis. Three independent experiments were carried out and a representative data set among them is shown here. Percentage of the fully and early apoptotic cells is presented in the upper and lower right quadrants, respectively. (**B**) Percentage of apoptotic and necrotic cells on MDR KB-V1 cells is plotted for each sample. The results represent the mean ± s.d. (n = 3). The percentage of apoptosis includes both the fully and early apoptotic cells.

Based on FACS analysis, we evaluated induction of apoptosis in the MDR KB-V1 cells when treated with various RNA nanoparticles 24 h prior to doxorubicin administration. A representative data set among three independent experiments is shown in the Fig. 7A. Doxorubicin alone, as a control, induced apoptosis to 11.3%, and necrosis to 2.9% (Fig. 7B). First, siBCL2 RNPs combined with doxorubicin was able to induce apoptosis to 17.5%, including both the fully and early apoptotic cells, and necrosis to 6.6%, while siMDR1 RNPs combined with doxorubicin could induce apoptosis to 23.5% and necrosis to 3.1%. However, Dsi RNPs combined with doxorubicin increased apoptosis to 38.0% and cell necrosis to 6.5%, which clearly shows that the simultaneous silencing of both MDR1 and BCL2 genes more efficiently enhances the cytotoxicity of drugs in MDR cells than the single silencing of either MDR1 or BCL2 gene. As expected, the degree of apoptosis and necrosis induced by the combination of the scrambled Dsc RNPs and doxorubicin was quite similar to that by doxorubicin alone, and also the scrambled Dsc RNPs alone did not induce any detectable apoptosis.

### Enhanced cytotoxicity of doxorubicin on MDR cancer cells after Dsi-24h-Dox-24h sequential treatment

**Figure 8:**
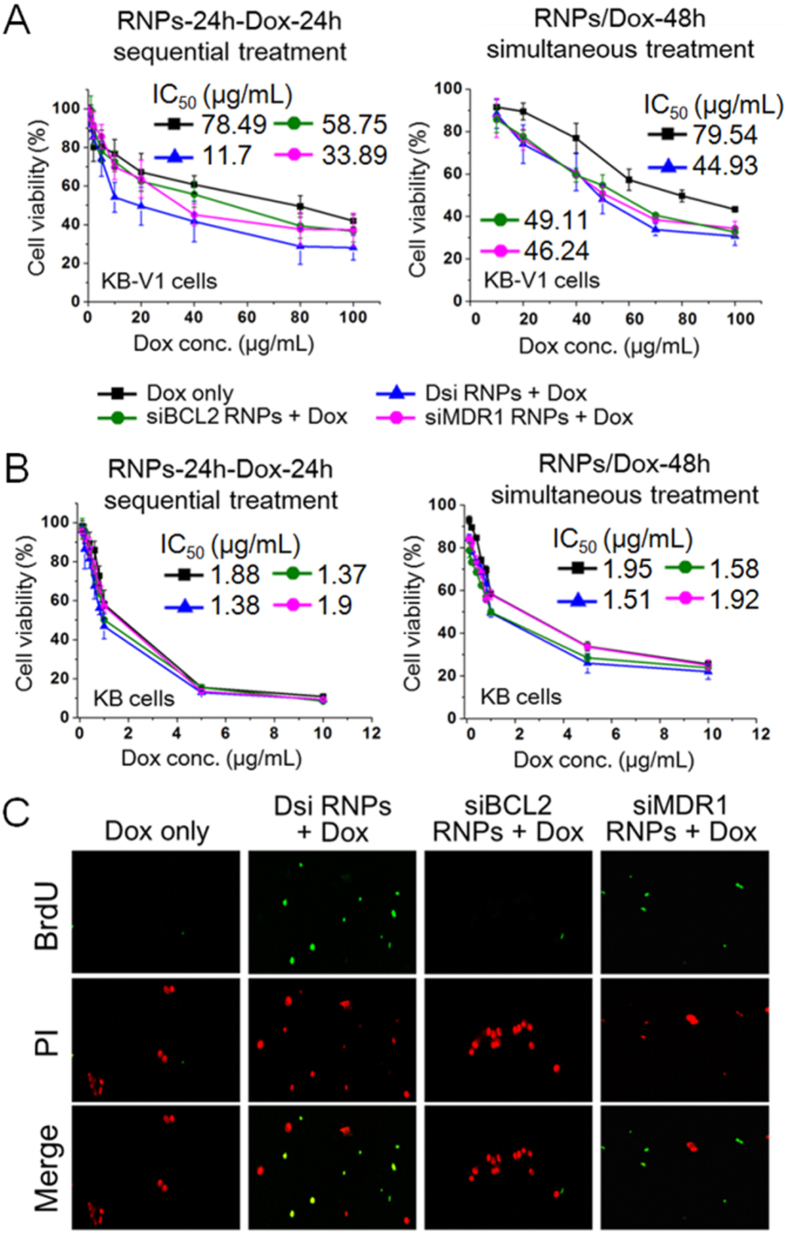
Chemosensitivity of MDR KB-V1 cancer cells to doxorubicin in the presence of various RNA nanoparticles. (**A**) Inhibition of cell growth of drug-resistant KB-V1 cancer cells treated in the RNPs-24h-Dox-24h method or RNPs/Dox-48h method. Various RNA nanoparticles (20 μg/mL) were treated, either alone or together with doxorubicin, in the two-step RNPs-24h-Dox-24h sequential or RNPs/Dox-48h simultaneous method, while doxorubicin was administered over a wide range of concentrations. 24 h after doxorubicin treatment, Cytotoxicities of doxorubicin were measured by using XTT assay. IC_50_ values of doxorubicin were calculated by Origin 8 using nonlinear regression analysis. Data were collected from 5 independent experiments and represented as mean ± s.d. (**B**) Inhibition of cell growth of KB cancer cells treated in the RNPs-24h-Dox-24h method or RNPs/Dox-48h method. Dsi RNPs-treated KB cells do not reveal any marked enhancement to the chemosensitivity in either two-step sequential or simultaneous method. (**C**) TUNEL assay of MDR KB-V1 cancer cells after RNPs-24h-Dox-24h treatment. DNA breaks and total cellular DNA content were visualized by Alexa Fluor 488-labeled anti-BrdU antibodies (green) and Propidium Iodide (red), respectively.

On the cell cytotoxicity studies using XTT assay, IC_50_ values of doxorubicin on MDR KB-V1 cell line and their KB parent cell line were determined as 78.49–79.54 μg/mL and 1.88–1.95 μg/mL respectively (Fig. 8A). Given that IC_50_ value is used as criteria of the cellular chemosensitivity to drugs, such a significant difference in IC_50_ values shows that the KB-V1 cell line has much higher chemoresistance to doxorubicin than the KB cell line.

To investigate whether RNA RNPs effectively suppress such chemoresistance, we measured IC_50_ value of doxorubicin in the combination therapy with various RNA RNPs. When each of siMDR1 RNPs and siBCL2 RNPs were pretreated to MDR KB-V1 cells in the two-step sequential method (siMDR1-24h-Dox-24h or siBCL2-24h-Dox-24h), IC_50_ value of doxorubicin was determined as 33.89 μg/mL and 58.75 μg/mL respectively, which corresponds to a 2.3-fold decrease and a 1.3-fold decrease. However, the Dsi-24h-Dox-24h sequential treatment (Dsi-24h-Dox-24h) exhibited 11.7 μg/mL of IC_50_ for doxorubicin, which corresponds to a 6.8-fold decrease in IC_50_ value. These results demonstrate that the simultaneous silencing of both genes, mediated by Dsi RNPs, can promote the chemosensitivity of MDR cells more efficiently than a separate single silencing. Meanwhile, a higher dosage of RNA nanoparticles, ranging from 20 μg/mL to 60 μg/mL, did not show any difference in IC_50_ values for doxorubicin (data not shown), and these data indicate that 20 μg/mL of RNA nanoparticles could effectively suppress such chemoresistance.

When Dsi RNPs and doxorubicin were simultaneously treated to MDR KB-V1 cells as in the Dsi/Dox-48h method, IC_50_ value of doxorubicin was measured as 44.93 μg/mL, corresponding to a 1.8-fold decrease. In each simultaneous treatment including the siMDR1/Dox-48h and siBCL2/Dox-48h treatment, IC_50_ value of doxorubicin was determined as 46.24 μg/mL and 49.11 μg/mL respectively, corresponding to a 1.7-fold decrease and a 1.6-fold decrease. Consequently, the combination therapy exploiting the simultaneous method failed to demonstrate that Dsi RNPs were far superior to siMDR1 RNPs or siBCL2 RNPs for promotion of chemosensitivity of MDR cancer cells. It is important to note that Dsi RNPs should be administered to MDR cancer cells in the two-step sequential method, not in the simultaneous method, to overcome the drug resistance. Interestingly, we did not observe any marked enhancement of chemosensitivity on the KB cells, either in the two-step sequential or simultaneous method (Fig. 8B). The scrambled Dsc RNPs did not affect the cytotoxicity of doxorubicin on either KB cells or KB-V1 cells, as expected (Supplementary Fig. S8).

On the other hand, calculation of combination index (CI), for combination therapy of MDR KB-V1 cells with Dsi RNPs and doxorubicin, indicated that the Dsi-24h-Dox-24h sequential treatment could achieve a synergistic effect (0.66 for CI <1). Thus, these results demonstrate that the simultaneous dual silencing through Dsi RNPs treatment can synergistically suppresses MDR KB-V1 cancer cells in the combined therapy with doxorubicin.

In the TUNEL assay for labeling DNA breaks, the Dsi-24h-Dox-24h treatment generated a high level of TUNEL-positive KB-V1 cells, whereas either the doxorubicin only or siBCL2-24h-Dox-24h treatment did not (Fig. 8C). The siMDR1-24h-Dox-24h treatment also generated the apoptotic cells, but the fraction of TUNEL-positive cells was much lower than that of the Dsi-24h-Dox-24h treatment. These results support that apoptotic changes, induced by the combined treatment of Dsi RNPs and doxorubicin, are closely associated with DNA fragmentation.

## Discussion

As an effort to achieve the simultaneous two-in-one siRNA delivery system, we demonstrate that our versatile RNA nanotechnology platform enables the production of multiple copies of two RNAi molecules within the same RNA nanoparticles. This method is capable of densely packing and co-delivering polymeric RNAi molecules, leading to the simultaneous silencing of two targeted mRNAs, of which biological functions are highly interdependent. Since DNA template is readily modified to encode the other specific RNAi molecules for two types of genes, this platform method can expand the applicability and versatility to elicit numerous therapeutic responses. Recently, we could succeed in synthesizing the “three-in-one” RNA NPs that contained multiple tandem copies of three different RNAi molecules, by encoding three different RNAi sequences with one DNA template *vi*a RCT reaction (unpublished works). These results enable us to anticipate further expanding the versatility of this platform method for regulating multiple gene functions. The one-step condensation/folate-displaying process allows the polymeric RNAi molecules to self-assemble into the more compact nanoparticles, when compared with the previously reported RNA nanoparticles that were generated by two-steps, separate condensation and ligand-displaying. Thus, ease of cargo loading for two or more types of siRNA has the potential as a practicable tool to achieve the simultaneous multiple gene silencing.

Our RNA nanotechnology platform allows a ratio of 1:1 of the different RNAi sequences to be readily incorporated into single chain of RNA polymers, because T7 RNA polymerases can achieve a process of unidirectional nucleic acid replication while they are rolling many times through the circular DNA template. High molecular weights of Dsi RNA transcripts, as shown on the gel retardation assay (Fig. 2A), indicate that RCT reaction proceeded without interruption and led to the generation of multiple tandem copies of the different RNAi sequences. Interestingly, if the circular DNA template is designed to contain two units of shRNA sequences (A type) and one unit of shRNA sequence (B type), a different ratio of RNAi types, such as a ratio of 2:1 of the different RNAi’s, would be readily achieved. In addition, a mixture of RCT reaction products, if obtained after RCT reaction for a 1:1 DNA template and a 2:1 DNA template respectively, would generate a ratio of 3:2 of the different RNAi’s *via* ultrasonication process for shuffling.

To circumvent the drug-resistant phenotypes of cancer cells, small compounds such as verapamil^35^, promethazine^36^, and cyclosporine A^37^, have been used as functional inhibitors, but currently, there is no drug that is clinically approved due to their undesirable pharmacokinetic behaviors and toxicities^38^. siRNA-based therapeutics, as an alternative approach, has been introduced and shown to successfully silence the drug-resistant phenotypes^34^,^39^. Recently, a combination of traditional chemotherapy with siRNA-based gene silencing has attracted great attention. For instance, polymer micelles simultaneously delivering doxorubicin (Dox) and BCL2 siRNA showed the great cell death effect on SKOV3 cancer^40^. Also, other various examples, including mesoporous silica nanoparticles^41^, gold nanorods^42^, carbonate–apatite nanoparticles^43^, and CdSe/ZnSe QDs^44^, have shown to enhance the chemosensitivity of MDR cancer cells by simultaneously delivering anticancer drugs with siRNA. However, there has still remained a substantial weakness in the “sole RNAi suppressor” strategies choosing a single type of RNAi molecule together with anticancer drugs, in that the silencing of only one type of resistance, either pump or non-pump, is not sufficient to overcome the development of chemoresistance in response to the cancer treatments^39^,^45^. Particularly, RNAi suppression of drug efflux pump causes a rapid increase of cellular drug concentration and thus proportionally triggers the significant activation of cell death defense system^46^, leading to an unfavorable therapeutic effect in MDR cancer. Therefore, it is important to note that our two-in-one RNAi delivery system provides an alternative approach to elicit the synergistic suppression of chemoresistance. However, there still remain challenges to precisely control the administrations of drugs and Dsi RNPs *in vivo* before the clinical applications, because Dsi RNPs are required to be administered earlier than anticancer chemotherapeutics for the best synergistic cancer suppression.

The combination therapy of Dsi RNA nanoparticles, together with chemotherapeutics, shows that their efficacy of silencing for both the pump and non-pump phenotypes does not diminish, when compared with that of a single RNAi-containing RNA nanoparticles, either siMDR1 RNPs or siBCL2 RNPs. With the aid of dicer enzymatic degradation, the release of both RNAi molecules from Dsi RNPs was precisely controlled in the cellular level. Dsi RNPs exhibit the selective and efficient intracellular delivery to the FR-positive KB-V1 cancer cells, while lowering the cellular uptake on the FR-negative cells, and therefore this distinct ability may lower the risk of severe adverse side effects, expected from the exceptionally increased cytotoxicity of siRNA-Dox combination. Although this active targeting strategy looks intriguing, it is worth noting that a limited fraction of Dsi RNPs can have the opportunity to interact with non-cancer cells, because the active targeting ability of Dsi RNPs exploits the relative properties between cancer cells and non-cancer cells based on the difference in the folate receptor expression levels. Meanwhile, when 10% of the cells in a breast tumor sample are stained for HER2/neu, these patients are recommended for Herceptin^®^ therapy, according to the FDA guideline^47^.

The best synergism expected from the combination therapy is closely associated with the precisely controlled timings at which siRNA and anticancer drugs separately function inside the same cancer cell. Unfortunately, most of the previous or current studies of combination therapy have reported the uncontrolled function of siRNA and drugs within cancer cells, which means that the drugs are penetrated into the cancer cells before the resistance is suppressed. To our knowledge, an ideal delivery carrier that meets such requirements has not been developed until now. To implement two-step sequential treatment (Dsi-24h-Dox-24h) *in vivo*, Dsi RNPs and doxorubicin need to be separately injected into body while maintaining the intervals of time (*e.g*. one day), and consequently the drug resistance phenotypes should be silenced before doxorubicin is unloaded at tumor sites. Therefore, a further effort to find out the optimal interval of time between Dsi RNPs and doxorubicin is required for the synergistic effects *in vivo*.

In contrast to traditional cationic polymeric vectors such as polyethylenimine (PEI) and poly-L-lysine (PLL) exploited for condensation of oligonucleotides^48^, Dsi RNPs do not induce any severe cytotoxicity on the cell viability studies (Supplementary Fig. S8), and moreover, do not stimulate any innate immune response in the human PBMCs (Supplementary Fig. S4). One of the major limitations found in systemic administration of RNAi therapeutics is due to their nonspecific uptake during blood circulation. Particularly, the mononuclear phagocyte system (MPS), which includes dendritic cells, monocytes, and macrophages responsible for degrading foreign materials from blood circulation, causes the opsonization and sequestration of RNAi therapeutics after intravenous administration^49^. In addition, MPS uptake can lead to inflammation and autoimmunity in the body, resulting in rapid clearance of RNAi therapeutics. Because this MPS process is affected by several factors such as particle size, surface charge, hydrophobicity, and surface chemistry, it is noticeable that the distinct characteristics of RNA nanoparticles are required to survive such immune responses. Positively charged particles tend to be readily sequestered by macrophages in the lung, liver and spleen^50^, whereas negatively charged RNA nanoparticles may not. In addition to no stimulatory properties for innate immunity, as shown in cytokine release studies, the highly enhanced stability of Dsi RNPs against serum nucleases, as a result of the densely packing, satisfies such the requirement during blood circulation. Herein, our RNA nanoparticles have great potential as the delivery system for multiple types of RNAi and our RNAi-based therapeutic approach, shown as a specific example of their biomedical applications, facilitates the synergistic therapy for MDR cancer.

## Methods

### Materials

T7 promoter primer, amino-cholesteryl (5′- and 3′-)-conjugated DNA, 3′-Cy5 labeled DNA, monomeric siRNA, and primers for qRT-PCR were all obtained from Bioneer Co. (Korea), as well as 5′-phosphorylated ssDNA templates for RCT reaction of various RNA nanoparticles including Dsi RNPs. The branched PEI (25 kDa) polymer was purchased from Sigma-Aldrich (UK). All oligonucleotide sequences are listed in Table S1.

### Preparation of FA-DNA-CHOL conjugate

In brief, we attached folate ligands (FA) to amino-cholesteryl (5′- and 3′-)-conjugated DNA (DNA-CHOL) fragments by using EDC (1-Ethyl-3-(3-dimethylaminopropyl)-carbodiimide) coupling reaction according to our previously reported protocol^3^. First, we added 4 mM EDC and 10 mM Sulfo-NHS to DNA-CHOL fragments (10 mM) dissolved in 100 mM MES buffer (pH 6.0) plus 500 mM NaCl. After gentle vortexing for 15 m, we added β-mercaptoethanol to deactivate the residual EDC reagents. Next, we added a 10-fold molar excess of folate to the reaction mixture, and then left it for 3 h at 24 °C. Through 3K MWCO of Amicon ultra-centrifugal filter (Milipore), we removed the unreacted reagents while repeatedly washing with nuclease-free water, and could obtained FA-DNA-CHOL conjugates.

### Synthesis of Dsi RNA nanoparticles *via* rolling circle transcription

We obtained the various RNA transcripts from the corresponding ssDNA templates *via* RCT reaction following the previously reported procedure^3^. To generate the condensed Dsi RNA nanoparticles, we added FA-DNA-CHOL fragments to the resulting Dsi RNA transcripts (Dsi RNAtr) dissolved in 30 mM Tris-HCl (pH 7.8) buffer including 10 mM MgCl_2_, at a weight ratio of 1:0.3 (Dsi RNAtr/FA-DNA-CHOL) in a total volume of 20 μL. The resulting mixture was subjected to heat denaturation at 65 °C for 5 m, and subsequently, cooled down to 4 °C for base pairing between Dsi RNA transcripts and FA-DNA-CHOL. Using Amicon ultra-centrifugal filter (3K MWCO), we purified the Dsi RNAtr/FA-DNA-CHOL complexes while repeated washing with distilled water. In a similar way, we could synthesize various RNA nanoparticles including siBCL2 RNPs (composed of BCL2 shRNA and scrambled MDR1 shRNA), siMDR1 RNPs (composed of MDR1 shRNA and scrambled BCL2 shRNA), and Dsc RNPs (composed of both scrambled shRNAs for MDR1 and BCL2). For the fluorescence imaging studies, we generated the various Cy5-labeled RNA NPs by further base-pairing of RNA NPs with Cy5-labeled DNA fragment at a weight ratio of 1:0.2.

### Characterization of Dsi RNPs

Using Zetasizer Nano ZS (Malvern) and Zetasizer Nano software, we could determine each molecular weight of Dsi RNA transcripts, as a result of a Debye plot calculation. We measured the intensities of scattered light at a single angle, while serially diluting the samples with nuclease-free water. Dsi RNA transcripts had 1.72 × 10^4^ ± 358 kDa and therefore, the molecular weight of Dsi RNPs (1:0.3, w/w) was estimated as 2.24 × 10^4^ ± 698 kDa. The hydrodynamic diameter and zeta potential of RNA particles were also measured by Zetasizer Nano ZS. All samples were filtered by syringe filter (0.22 μm MWCO) before measurement and the data was collected at a laser wavelength of 633 nm with a scattering angle of 173°.

For the stability studies of RNA particles against serum nucleases, we added fetal bovine serum solution (30% at the final concentration) to Dsi RNA transcripts (10 μg/mL) or Dsi RNPs solution (10 μg/mL), and incubated the mixture according to the indicated time period. We electrophoresed each mixture on 15% non-denaturing TBE gel and quantitatively analyzed the band intensities by using a Gel Doc image analysis system (Bio-Rad). Other additional tools or methods for characterization of RNA NPs are described in the Supplementary Information.

### Dicer cleavage assay

We reacted human recombinant dicer enzyme (1.5 unit, Genlantis) with 5 μg of Dsi RNA transcripts or Dsi RNPs in the final volume of 20 μL at 37 °C. The reaction buffer was as follows; 4 mM Tris-HCl (pH 7.9), 10 mM ATP, 50 mM MgCl_2,_ 1 mM DTT and 0.2 mM spermidine. 2 μL of Dicer Stop Solution (Genlantis) was added to quench the reaction, and then each sample was electrophoresed on gel (3%).

### Stimulation assay for assessing innate immune activation

Human PBMCs were purchased from Astarte Biologics (Redmond, WA), and the donors were negative for HIV-1 and -2, HBV, HCV, and HTLV. PBMCs were isolated from healthy donors’ whole blood according to the previously reported procedure^51^. These studies on the use of human blood and tissue samples were performed and approved in accordance with the regulation of the Institutional Use Committee and Animal Care Committee of Korea Institute of Science and Technology (Ref. No. KIST 2016-043). Throughout the current studies, the informed consent was obtained from all subjects. We seeded the freshly isolated PBMC cells into 96-well plates at density of 2 × 10^5^ cells/well in RPMI 1640 supplemented with 10% FBS plus 2 mM L-glutamine. After the cells were completely attached, we stimulated them with various reagents as follows: monomeric siRNA (50 nM MDR1 siRNA plus 50 nM BCL2 siRNA), monomeric siRNA/PEI (branched, 25 kDa) (equivalent to 50 nM MDR1 siRNA plus 50 nM BCL2 siRNA), Dsi RNA transcripts (20 μg/mL), Dsi RNPs (26 μg/mL), and CpG ODN (5 μM) as a positive control of INF-α or resiquimod R-848 (10 μg/mL) as a positive control of IL-6. At 24 h post-treatment, we measured the released INF-α and IL-6 on the supernatants by using VeriKine™ Human INF-α ELISA Kit (PBL Biomedical) and Human IL-6 ELISA Ready-SET-Go Kit (eBioscience) following the manufacturer’s instruction.

### Cellular binding and uptake studies

To assess the FR-dependent cellular binding and uptake, FACS and confocal microscopic analysis were carried out on the FR-negative HepG2 and FR-positive KB-V1 cells. First, each cell lines were plated on 96-well plates at a density of 1 × 10^5^ cells/well and cultured to reach 70–80% confluency. After incubating the cells in the freshly prepared FBS-containing media for 30 m, we treated the Cy5-labeled Dsi RNPs or Cy5-labeled non-targeted Dsi RNPs to each cell lines at the final concentration of 20 μg/mL, and 3 h later, washed the cells three times with DPBS. The cellular binding of Cy5-labeled RNA nanoparticles was investigated by EasyCyte flow cytometry system (Guava Technologies) with the aid of a red laser (640 nm). Also, the cellular internalization was examined by LSM710 laser-scanning confocal microscope (Carl Zeiss). The high magnification images were measured with 63X objective and the 3D images were obtained by LSM710 ZEN software (Carl Zeiss). For clarity, nucleus was stained by DAPI dyes. To block folate receptors on the surface of cells, we treated 0.5 mM of free folate ligands 30 m prior to Dsi RNPs administration.

### Gene silencing studies

qRT-PCR was first carried out to investigate gene silencing efficacy. For comparison of gene silencing efficacy, various RNA nanoparticles (20 μg/mL) and doxorubicin (30 μg/mL) were treated on KB-V1 cells sequentially or simultaneously. As preliminary to qRT-PCR studies, IC_50_ of doxorubicin in MDR KB-V1 cells was determined as 80.05 μg/mL, which was consistent with that of the previous studies^32^, and also we could observe that 30 μg/mL of doxorubicin fully caused the multi-drug resistance in the MDR KB-V1 cells. For the two-step sequential treatment of RNA nanoparticles and doxorubicin (*that is*, RNPs-24h-Dox-24h sequential treatment), the cells were pretreated with RNA nanoparticles 24 h before doxorubicin administration. After each doxorubicin treatment, the cells were further incubated for 24 h. For the simultaneous treatment (*that is*, RNPs/Dox-48h simultaneous treatment), KB cells or KB-V1 cells were simultaneously treated with RNA nanoparticles (20 μg/mL) and doxorubicin (30 μg/mL), and then further incubated for 48 h. We isolated mRNA from each cell samples and then generated cDNA following RNeasy Mini Kit (Qiagen) and High Capacity RNA-to-cDNA kit (Applied Biosystems) protocols. After the cDNAs were mixed with 20 μL of reaction solutions including 2x SYBR Green mix (Applied Biosystems) and DNA primers, we carried out qRT-PCR by using a StepOnePlus real-time PCR systems (Applied Biosystems). For western blotting analysis, KB-V1 cells were treated with various RNA nanoparticles and doxorubicin in the aforementioned methods. Each cell lysates were isolated in RIPA buffer (Sigma) and immunoblotted with HRP-conjugated anti-BCL-2 monoclonal antibody (Santa cruz) or anti-P-Glycoprotein polyclonal antibody (Abcam) followed by HRP-conjugated goat anti-rabbit IgG (Abcam). The relative expression of BCL2 or P-gp proteins was normalized against expression of β-tubulin, which was detected with HRP-conjugated polyclonal anti-β tubulin antibody (Abcam). The western blotting images were measured by EZ-Capture MG (Japan) and quantified by Image J software. On the other hand, we did not observe any difference in the synergistic effect when increasing the concentration of RNA nanoparticles from 20 μg/mL to 60 μg/mL in the presence of a fixed amount of doxorubicin (30 μg/mL), and therefore, the combination of 20 μg/mL of RNA nanoparticles and 30 μg/mL of doxorubicin resulted in the best synergistic effects on the KB-V1 cells.

### Measurement of concentration of intracellular doxorubicin

KB-V1 cells were placed on a 35 mm confocal petri dish and treated with Dsi RNA nanoparticles (20 μg/mL) and Doxorubicin (30 μg/mL) in the two-step Dsi-24h-Dox-24h sequential treatment as mentioned above. The cells were washed three times with DPBS 3 h after doxorubicin treatment, and 9 h later, the fluorescence signals, emitted from the intracellular doxorubicin on KB-V1 cells, were examined by a Carl Zeiss LSM710 laser-scanning confocal microscope. To quantitatively measure concentration of the intracellular doxorubicin, we first plated KB-V1 cells on 96-well plates at a density of 2 × 10^4^ cells/well, and then treated the cells with doxorubicin alone or with both RNA nanoparticles and doxorubicin in the aforementioned method. Next, we washed the cells three times using DPBS 3 h after doxorubicin treatment, and 9 h later, measured the fluorescence signals using a Xenogen IVIS Lumina imaging system (Perkin Elmer) and Image J software (NIH).

### Apoptosis assay

We assessed the induction of apoptosis on KB-V1 cells by using Annexin V-FITC/PI kit (BD Pharmingen). After treating 2 × 10^5^ of KB-V1 cells with Dsi RNPs (20 μg/mL) and doxorubicin (30 μg/mL) in the aforementioned two-step Dsi-24h-Dox-24h method, we harvested the cells, and subsequently stained them with Annexin V-FITC/PI solution for 15 m at room temperature. We added 0.1 M HEPES buffer (7.4) containing 1.4 M NaCl and 25 mM CaCl_2_, and then carried out FACS analysis. Percentage of the fully and early apoptotic cells is presented as the mean value of three independent experiments, and a representative FACS histogram is shown. To more validate the cellular apoptosis, we performed TUNEL staining assay. After treating 2 × 10^5^ of KB-V1 cells with various RNA NPs (20 μg/mL) and doxorubicin (30 μg/mL) in the aforementioned two-step RNPs-24h-Dox-24h method, we trypsinized the cells, and then centrifuged them. The obtained pellets were washed with PBS buffer and fixed in 1% (w/v) paraformaldehyde solution for 15 m on ice. The cells were washed several times with PBS, and then permeabilized with 70% ethanol for 30 m on ice. As a labeling step, the cells were reacted with DNA-labeling solution containing TdT enzyme and Br-dUTP at 37 °C for 60 m (APO-BrdU^TM^ TUNEL assay kit, Invitrogen). Next, the cells were washed and stained with fluorescence dye-labeled anti-BrdU antibodies for 30 m, and then treated with propidium iodide and RNAse A. Propidium iodide as a control was used to visualize the total cellular DNA content. We obtained the fluorescence images by using a Carl Zeiss LSM710 laser-scanning confocal microscope. Three independent experiments were carried out for statistical analysis.

### Cell cytotoxicity assay

We examined the cytotoxicities of doxorubicin, either alone or in the presence of various RNA NPs, on the drug-resistant KB-V1 cell line and their KB parent cell line. In brief, each cell lines seeded on 96-well plates (2 × 10^4^ cells/well) were incubated with FBS-containing media for 30 m. Various RNA nanoparticles were treated at the final concentration of 20 μg/mL, either alone or together with doxorubicin, in the two-step RNPs-24h-Dox-24h sequential or RNPs/Dox-48h simultaneous method, while doxorubicin was administered over a wide range of concentrations for 24 h. 24 h after doxorubicin treatment, we repeatedly washed the cells with 10% FBS-containing media, and then further incubated them for 1 day. Next, we added 100 μL of XTT solutions to each well, and then incubated the cells for 4 h in the dark condition. After collecting the supernatants, we measured the cell cytotoxicities by using XTT colorimetric assay (Promega) and a microplate reader (Spectra MAX 340, Molecular Devices).

## Additional Information

**How to cite this article**: Jang, M. *et al*. A RNA nanotechnology platform for a simultaneous two-in-one siRNA delivery and its application in synergistic RNAi therapy. *Sci. Rep.* **6**, 32363; doi: 10.1038/srep32363 (2016).

## Supplementary Material

Supplementary Information
