# Supplementary material for: A RNA nanotechnology platform for a simultaneous two-in-one siRNA delivery and its application in synergistic RNAi therapy
Source: Sci Rep. 2016 Aug 26;6:32363. doi: 10.1038/srep32363 (PMC4999871; doi:10.1038/srep32363)
Supplement: Supplementary Information [file srep32363-s1.pdf]

Supplementary Information

A RNA nanotechnology platform for a simultaneous two-in-one siRNA delivery and its application in synergistic RNAi therapy

Mihue Jang, Hee Dong Han, and Hyung Jun Ahn

Determination of conjugation ratio between folate and DNA-cholesterol. The conjugate ratio of folate to DNA-CHOL fragment could be determined by measuring the extinction coefficients of folate and DNA-CHOL fragment at 260 nm and 363 nm respectively, on UV/Vis spectrophotometer (Agilent); $A_{260} = \varepsilon_{260}(FA) \cdot [FA] + \varepsilon_{260}(DNA\text{-}CHOL) \cdot [DNA\text{-}CHOL]$, $A_{363} = \varepsilon_{363}(FA) \cdot [FA] + \varepsilon_{363}(DNA\text{-}CHOL) \cdot [DNA\text{-}CHOL]$. The standard curves were prepared at both wavelengths following the previous protocol[1]. The ratio of folate to DNA-CHOL fragment was determined as 0.93.

AFM, FE-SEM and TEM. Using atomic force microscopy (AFM) imaging (PSIA XE-100 AFM system, PSIA), we could measure the size of particles. First, 10 μL of sample was dispensed onto the freshly cleaved mica surfaces, and then air-dried at room temperature. AFM measurements were performed using a 100 μm scanner with tapping mode at a scan rate of 1 Hz, and AFM imaging was analyzed by XEI version 1.7. To examine the morphology of RNA nanoparticles, we acquired the FE-SEM images using field emission scanning electron microscopy (FESEM, Hitachi S-4100). Moreover, we measured the size and morphology of particles using CM30 electron microscope (Philips). After a carbon-coated copper grid was activated by mild plasma cleaning, one drop (10 μL) of sample (26 μg/mL) was placed on the grid. The grid was slowly air-dried at 22 °C. TEM images could be measured without the aid of staining.

Cell culture. HepG2 and KB cells were grown in RPMI1640 (WelGENE) and DMEM (WelGENE) including 10% FBS and 100 μg/mL penicillin-streptomycin. MDR KB-V1 cells were grown in DMEM supplemented with 15% FBS plus 0.5 μg/mL of vinblastine (Invivogen). All cell cultures were maintained at 70 - 80% confluency, changing medium every 2 - 3 days. MDR KB-V1 cancer cells and their KB parent cancer cells were obtained from DSMZ (Germany) and Korean Cell Line Bank (Korea), respectively. HepG2 cells were kindly provided by Dr. Kwangmyeung Kim, KIST, Korea.

Determination of loading efficacy of siRNA in Dsi RNPs. The loading efficacy of

functional siRNA duplex in Dsi RNPs was calculated as follows. Each Mw of Dsi RNA transcripts generated during a single replication cycle of RCT, functional BCL2 siRNA duplex, functional MDR1 siRNA duplex, and FA-DNA-CHOL conjugate are 57350.1 g mol^{-1}, 14555.6 g mol^{-1}, 13284.8 g mol^{-1}, and 7129.2 g mol^{-1}, respectively. Thus, the theoretical loading efficacy (w/w) of functional siRNA duplex in Dsi RNPs is 37.3%.; weight of functional BCL2 siRNA duplex plus MDR1 siRNA duplex / {weight of a single replication cycle of Dsi RNA transcripts/FA-DNA-CHOL (1:0.3, w/w)} x 100 (%) = (27840.4 g mol^{-1}) / (74555.1 g mol^{-1}) x 100 (%) = 37.3 (%). Based on the dicer cleavage assay, an experimental cleavage efficacy is 33.8%, and therefore, the dicer cleavage efficacy *in vitro* is 90.6%; experimental dicer cleavage yield / theoretical loading efficacy = 33.8 / 37.3 = 90.6 (%).

Calculation of combination index (CI). Combination index (CI) values were calculated by the following equation; CI = (Dose of Dsi RNPs_Dox_{IC50}/Dose of siBCL2 RNPs_Dox_{IC50}) + (Dose of Dsi RNPs_Dox_{IC50}/Dose of siMDR1 RNPs_Dox_{IC50}) + α[(Dose of Dsi RNPs_Dox_{IC50})(Dose of Dsi RNPs_Dox_{IC50})/(Dose of siBCL2 RNPs_Dox_{IC50})(Dose of siMDR1 RNPs_Dox_{IC50})], where α value was zero for the more conservative assumption of mutual exclusivity as shown in the previous studies[2]. CI value was calculated to examine whether the combination therapy may be synergistic (CI < 1), additive (CI = 1), or antagonistic (CI > 1).

Linear DNA template for Dsi RNPs	5'-ATAGTGAGTCGTATTAACGTACCAACAA*GACAGCTTATAATGGATGTAC*TTACTTGCCCGGAAACCCTGGT*GTACATCCATTATAAGCTGTC*GTATTAACGTACCAACAA*GACAGTTGGTTTCTTTTCC*TTACTTGCCCGGAAACCCTGGT*GGAAAAGAAACCAACTGTC*TTAGAGGCATATCCCT-3'
T7 promoter primer	5'-TAATACGACTCACTATAGGGAT-3'
FA-DNA-CHOL fragment	folate-5'-TACTTGCCCGGAAACCCTGG-3'-cholesterol
anti-MDR1 siRNA duplex	5'-GGAAAAGAAACCAACUGUC-3'<br>3'-CCUUUUCUUUGGUUGACAG-5'
anti-BCL2 siRNA duplex	5'-GUACAUCCAUUAUAAGCUGUC-3'<br>3'-CAUGUAGGUAAUAUUCGACAG-5'
Linear DNA template for siMDR1 RNPs	5'-ATAGTGAGTCGTATTAACGTACCAACAAATGAGTGCATAGATTTCAAGCTTACTTGCCCGGAAACCCTGGTGCTTGAAATCTATGCACTCATGTATTAACGTACCAACAA*GACAGTTGGTTTCTTTTCC*TTACTTGCCCGGAAACCCTGGT*GGAAAAGAAACCAACTGTC*TTAGAGGCATATCCCT -3'
Linear DNA template for siBCL2 RNPs	5'- ATAGTGAGTCGTATTAACGTACCAACAA*GACAGCTTATAATGGATGTAC*TTACTTGCCCGGAAACCCTGGT*GTACATCCATTATAAGCTGTC*GTATTAACGTACCAACAACCGTATCGTAAGCAGTACTTTACTTGCCCGGAAACCCTGGTAGTACTGCTTACGATACGGTTAGAGGCATATCCCT-3'
Linear DNA template for scrambled Dsc RNPs	5'- ATAGTGAGTCGTATTAACGTACCAACAAATGAGTGCATAGATTTCAAGCTTACTTGCCCGGAAACCCTGGTGCTTGAAATCTATGCACTCATGTATTAACGTACCAACAACCGTATCGTAAGCAGTACTTTACTTGCCCGGAAACCCTGGTAGTACTGCTTACGATACGGTTAGAGGCATATCCCT -3'
MDR1 forward primer	5'-GAAATTTAGAAGATCTGATGTCAAACA-3'
MDR1 reverse primer	5'-ACTGTAATAATAGGCATACCTGGTCA-3'
BCL2 forward primer	5'-AGTACCTGAACCGGCACCT-3'
BCL2 reverse primer	5'-GCCGTACAGTTCCACAAAGG-3'
β-actin forward primer	5'-AGAGGGAAATCGTGCGTGAC-3'
β-actin reverse primer	5'-CAATAGTGATGACCTGGCCGT-3'

Table S1. Oligonucleotide sequence information in the current studies. The blue sequences of Dsi RNA transcripts, as a result of RCT reaction, are designed to be complimentary to the blue sequences of FA-DNA-CHOL fragment, and consequently, are involved in base pairing with FA-DNA-CHOL fragments. Each colored italic sequences of Dsi RNA transcripts are designed to contain the same colored sequences of sense and antisense strands of either anti-MDR1 siRNA (green) or anti-BLC2 siRNA (red). The black sequences of Dsi RNA transcripts are complimentary to partial sequences of T7 promoter

primer. The underlined sequences of linear DNA template correspond to the scrambled sense/antisense sequences of either anti-MDR1 siRNA or anti-BLC2 siRNA.

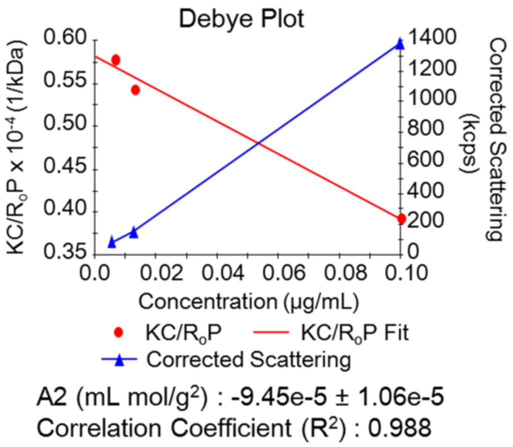

Figure S1. Debye plot for determination of Mw of Dsi RNA transcripts. Based on a Debye plot from SLC, MW of Dsi RNA transcripts could be estimated from intercept at zero concentration.

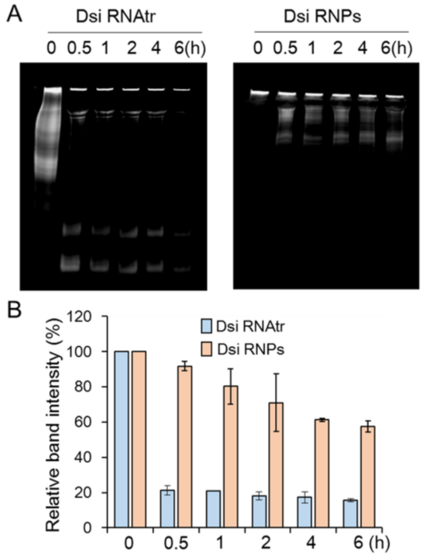

Figure S2. Stability studies of RNA transcripts (Dsi RNAtr) and RNA nanoparticles (Dsi RNPs) in FBS solution. (A) Dsi RNAtr and Dsi RNPs incubated in 30% FBS solution for the time indicated. Each sample was electrophoresed on 15% TBE gel. (B) The relative intensities of bands plotted according to the incubation time. The results are shown as the mean ± s.d. (n=5).

A

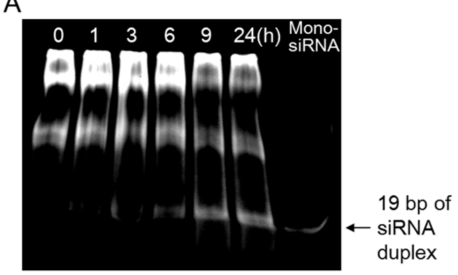

B

	Band intensity (AU)	Amount (μg)
19 bp of mono-siRNA duplex as a standard	4601.9	0.5
Short RNA generated from Dsi RNPs	15646.6	1.69

Figure S3. Conversion of Dsi RNPs into siRNAs by dicer cleavage *in vitro*. (A) 5 μg of Dsi RNPs incubated with recombinant human dicer enzyme (1.5 U) for the periods indicated. Each sample was electrophoresed on 15% TBE gel. (B) Based on 19 bp of monomeric siRNA duplexes (0.5 μg) as a reference, the amount of short RNA strands, converted from Dsi RNPs, was estimated as 1.69 μg.

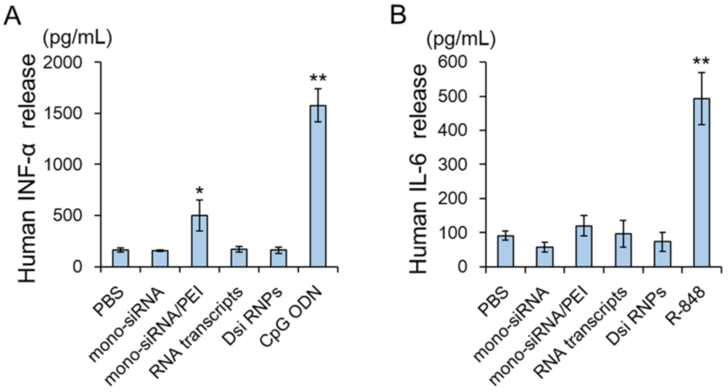

Figure S4. Proinflammatory cytokine induction of Dsi RNPs on human PBMCs. Induction of INF-α release (A) and IL-6 (B) on the human PBMCs. INF-α release or IL-6 release was examined 24 h after various stimuli as follows: PBS, Mono-siRNA (50 nM MDR1 siRNA plus 50 nM BCL2 siRNA), Mono-siRNA/PEI (equivalent to 50 nM MDR1 siRNA plus 50 nM BCL2 siRNA), Dsi RNAtr (20 μg/mL), or Dsi RNPs (26 μg/mL). 5 μM CpG ODN and 10 μg/mL R-848 were treated as the positive control for INF-α and IL-6 release, respectively. The results are shown as the mean ± s.d. (n=5). * $p < 0.05$, ** $p < 0.001$ by one-way ANOVA with Tukey`s multiple comparison test, as compared to the PBS control.

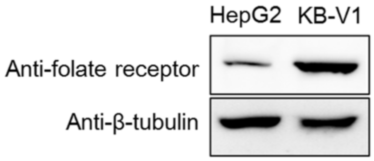

Figure S5. Immunoblotting analysis of folate receptors on FR-negative HepG2 liver carcinoma cells and FR-positive KB-V1 cervix carcinoma cells. Each cell lysates were immunoblotted with HRP-conjugated anti-folate receptor antibodies followed by HRP-conjugated goat anti-rabbit IgG. The relative expression of folate receptors was normalized relative to the expression of β-tubulin. The western blotting images were measured by EZ-Capture MG.

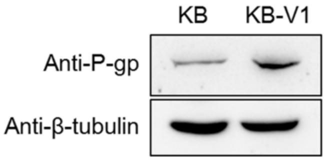

Figure S6. Immunoblotting analysis of P-gp proteins on drug-resistant KB-V1 cell line and their KB parent cell line. Each cell lysates were immunoblotted with HRP-conjugated anti-P-Glycoprotein polyclonal antibody followed by HRP-conjugated goat anti-rabbit IgG. The relative expression of folate receptors was normalized relative to the expression of β-tubulin.

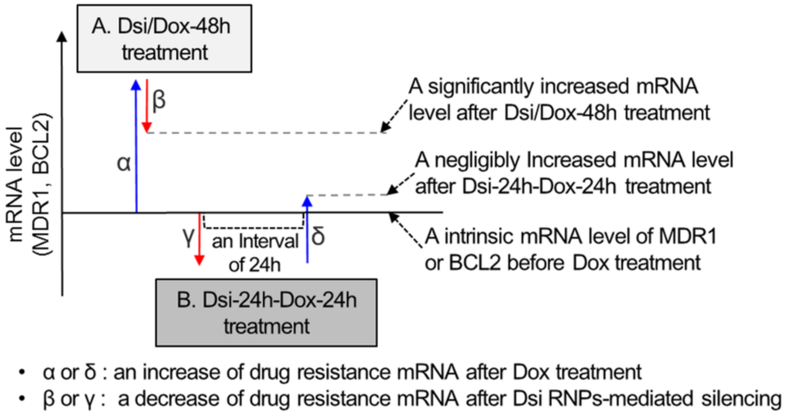

Figure S7. Schematic diagram demonstrating activation of drug resistance genes induced by doxorubicin. The Dsi/Dox-48h simultaneous treatment shows that rapid activation of MDR1 and BCL2 gene, which is induced by the function of doxorubicin before Dsi RNPs trigger mRNA degradation, leads to a failure in suppression of both MDR1 and BCL2 mRNAs even in the presence of Dsi RNPs (A). In contrast, the two-step Dsi-24h-Dox-24h sequential treatment allows sufficient time to degrade both mRNA before doxorubicin induces the activation of both genes, and consequently, succeeds in suppressing robust activation of both genes (B). Thus, doxorubicin needs to be administered to drug-resistant cancer cells "some time" after Dsi RNPs treatment.

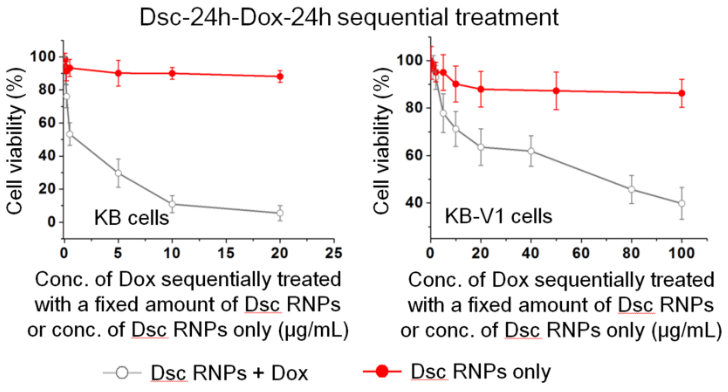

Figure S8. Chemosensitivity of MDR KB-V1 cancer cells to doxorubicin in the presence of scrambled RNA nanoparticles. Inhibition of cell growth of drug-resistant KB-V1 cells or their KB parent cells treated in the Dsc-24h-Dox-24h method. Cytotoxicities of doxorubicin in the presence of scrambled RNA nanoparticles (20 μg/mL) were measured by using XTT assay. IC_{50} values of doxorubicin were calculated by Origin 8 using nonlinear regression analysis. In addition, cell viability data of Dsc RNA nanoparticles alone in the absence of doxorubicin show that Dsc RNA nanoparticles are not severely cytotoxic in either KB cells or MDR KB-V1 cells. Similarly, Dsi RNA nanoparticles alone in the absence of doxorubicin did not induce any severe cytotoxicity on the XTT assay (data not shown). Data were collected from 5 independent experiments and represented as mean ± s.d.

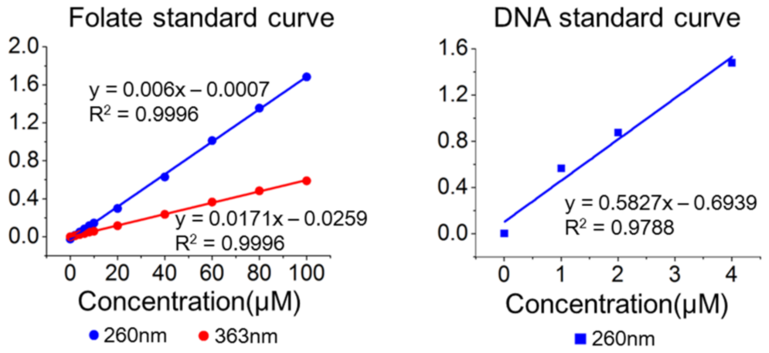

Figure S9. Standard curves for determination of a conjugation ratio between folate and DNA-CHOL fragment. Extinction coefficients were determined for folate and DNA-CHOL fragment by using standard curves at 260 and 363 nm. The conjugation ratio of folate to DNA-CHOL on FA-DNA-CHOL fragment was calculated as 0.93.

References

1. Vlashi, E., Kelderhouse, L. E., Sturgis, J. E. & Low, P. S. Effect of folate-targeted nanoparticle size on their rates of penetration into solid tumors. *ACS Nano* **7**, 8573-8582 (2013).
2. Yu, J., Drisko, J. & Chen, Q. Inhibition of pancreatic cancer and potentiation of gemcitabine effects by the extract of Pao Pereira. *Oncol. Rep.* **30**, 149-156 (2013).
